# Fat grafting and platelet-rich plasma in wound healing: a review of histology from animal studies

**DOI:** 10.1080/21623945.2021.1876374

**Published:** 2021-02-02

**Authors:** Grant S. Nolan, Oliver J. Smith, Gavin Jell, Afshin Mosahebi

**Affiliations:** Division of Surgery & Interventional Science, University College London, London, UK

**Keywords:** Adipose-derived stem cells, fat grafting, platelet-Rich Plasma, wound healing, diabetic foot ulcer, mesenchymal stem cells

## Abstract

Stem cells could form the basis of a novel, autologous treatment for chronic wounds like diabetic foot ulcers. Fat grafts contain adipose-derived stem cells (ADSC) but low survival of cells within the grafts is a major limitation. Platelet-rich plasma (PRP) may increase graft survival. This review examines the histology from animal studies on fat grafting, ADSC and PRP in wound healing. A literature review of major electronic databases was undertaken, and narrative synthesis performed. Data from 30 animal studies were included. ADSC increase angiogenesis over 14 days and often clinically accelerated wound healing. ADSC had a greater effect in animals with impaired wound healing (e.g. diabetes). Activated PRP increased viability of fat grafts. Despite the high number of studies, the quality is variable which weakens the evidence. It does suggest there is a benefit of ADSC, particularly in impaired wound healing. High-quality evidence in humans is required, to establish its clinical usefulness.

## Introduction

Chronic wounds, such as diabetic foot ulcers, are breaks in the skin that do not progress through the normal stages of wound healing in a timely manner[1]. They cost the United Kingdom National Health Service (NHS) over £580 million each year[2], affect patients’ employment and independence, and may progress to gangrene that requires amputation. Diabetic foot ulcers are the leading cause of lower limb amputations as 84% of patients have a preceding diabetic foot ulcer[3]. Diabetes reduces wound healing capability through prolonged inflammation, reduced growth factors production, and impaired angiogenesis amongst others[4]. Currently, the treatment of diabetic foot ulcers is unsatisfactory and consists of podiatry care, vascular optimization, and preventing infections. Consequently, there is an urgent need for new approaches. The administration of stem cells and growth factors could form the basis of a novel treatment, which could restore the body’s normal healing processes.

Adipose-derived stem cells (ADSC) are adult multipotent, mesenchymal stem cells located in adipose tissue [5] that have recently become popular in the field of regenerative medicine for cell-based therapies. ADSC can undergo self-renewal, differentiate into cell types involved in skin repair, and also secrete many growth factors themselves[6]. They are easily harvested via liposuction in large quantities with very little donor site morbidity. Additionally, ADSC are far more abundant than other forms of mesenchymal stem cells like those found in bone marrow (ADSC can comprise up to 1% of fat compared to 0.001%–0.002% of cells in bone marrow) [7]. All of these factors make ADSC appealing candidates for accelerating wound healing. This would be achieved through ‘fat grafting’, where the lipoaspirate containing ADSC and adipocytes is injected or topically applied to the chronic wounds. United Kingdom (UK) legislation currently prevents the use of stem cells in clinical practice so fat grafting without further processing is considered the next best alternative. It is clinically already widely utilized by plastic surgeons for soft tissue reconstructions and in aesthetic procedures[8].

The low survival rate of cells within fat grafts hinders their use in all settings. The proportion of surviving cells within grafts was reported in a systematic review of ten in vivo studies as 15% to 58% over 1 to 12 months[9]. The methods used in some the included studies to calculate the percentage may be prone to error, through individual variation and lack of repeatability due to the techniques used, such as dissection of the graft and weighting [10,11]. In the systematic review [9] only one study used 3d imaging for objective measurements of graft volume [12] and they reported the survival rate was only 18%. Additional factors such as the anatomical layer the fat is grafted can also affect the long-term viability and volume of fat graft surviving (graft survival of: 81% in supra-muscular layer; 37% submuscular layer; 41% in subcutaneous tissue)[13].

Ischaemia is hypothesized as the main cause of adipocyte death in the fat grafts which can make cells non-viable within 24 hours[14]. Combining the fat grafts with platelet-rich plasma (PRP), an autologous blood product and potent source of growth factors, may increase re-vascularization of the fat grafts and enhance the proportion of cells surviving. Besides early re-vascularization, three other hypotheses have been proposed in the literature for how PRP may further potentiate the survival of ADSC and fat grafts.

1. PRP may enhance the proliferation and differentiation of ADSC[15].. If PRP caused ADSC to proliferate more, then these new cells could replace dying cells enabling a greater population of cells to participate in wound healing. If PRP causes ADSC to differentiate into relevant cell lineages, then these new cells could be directly involved in tissue repair. Previously it has been shown in vitro that PRP causes ADSC differentiation into fibroblasts and keratinocytes [16] which are key cell types for wound healing.
2. Fibrin within the PRP could enhance cell survival through a reduction in anoikis[17].
3. PRP may increase the secretion of pro-angiogenic growth factors from ADSC [18] which would increase graft re-vascularization.

PRP is manufactured through the centrifuging of blood and is defined as plasma with an elevated platelet concentration[19]. When activated (with calcium chloride or thrombin) there is a rapid and un-coordinated release of the α-granules from within platelets [20] which contain growth factors that are key mediators in angiogenesis and cellular proliferation such as vascular endothelial growth factor (VEGF), fibroblast growth factor (FGF) and transforming growth factor β (TGF-β) amongst others [19,21]. α-granules also contain cytokines and adhesion molecules[22]. PRP is autologous so is safe when re-injected.

The aim of this review was to examine the histology evidence from animal studies on fat grafting, ADSC and PRP in the context of wound healing. The knowledge from this review will shape future research and may facilitate the development of novel, autologous treatments for wound healing, which would be particularly useful in chronic wounds like diabetic foot ulcers.

## Materials and methods

A literature review was undertaken of two major databases: MEDLINE and Embase were searched using a broad range of terms and keywords related to fat grafting, ADSC, PRP, and wound healing. All comparative studies reporting histology changes in cutaneous tissue following the application of ADSC, fat grafting or PRP were included. Case reports or series with less than 3 animals were excluded, as were reviews and letters. One reviewer undertook the searches, and the references of included studies underwent hand searching for other potentially relevant material. The search was performed in English and non-English articles were not included. The primary outcome was changed in histology following application of fat grafts, ADSC or PRP. Secondary outcomes included the clinical changes observed in wound healing, and the stains used.

The following data were extracted: study demographics; number and species of animal models used including any treatments to simulate disease such as diabetes; full details of the intervention for ADSC and PRP (including methods of activation); control details; methods of examining wound healing including specific histological and immunohistology stains used; histological and immunohistological results; clinical results. Three study authors [23–25] were contacted about further data however no reply was received. The data were synthesized into a narrative review.

## Results

Following searching, 30 animal studies [10,11,23–50] met inclusion criteria and underwent data extraction and narrative synthesis. A summary of the results is shown in Table 1. The complete results tables of all included studies with extracted data are available as supplementary material.

**Table 1.** Summary of results from different animal studies in terms of the two commonest outcomes: angiogenesis and clinical changes

	Host	Outcome	
		Angiogenesis	Clinical
Adipose-derived stem cells (ADSC)	Animals with impaired wound healing	At 7 days: Increased agenesis by 70–106% in all 7 studies^[23–25,31–34]^ From day 8 onwards: Continued increased in angiogenesis compared to negative controls^[23,31–34]^ No difference^[25]^	Accelerated wound healing in al 7 studies^[23–25,31–34]^
	Healthy animals	At 7 days: Increased vessel density by 66–104% in 3 studies^[25,35,36]^ No difference in 2 studies^[31,33]^ From day 8–14: Increased angiogenesis but less than in animals with impaired wound healing in 8 studies^[23,25,26,28,31–34]^ Day 14 onwards: No difference in 2 studies^[29,30]^	Accelerated wound healing in 8 studies^[24–26,28–30,35,36]^ No difference in 1 study^[27]^
Platelet-rich plasma (PRP)	Healthy animals	Increased angiogenesis by 60–260% in 5 studies^[38,40–44]^ Not reported/not increased in 2 studies^[37,39]^	Accelerated wound healing in 5 studies^[40–44]^ No difference in 1 study^[39]^

### Methods used to examine wound healing histology

Included studies use two different approaches to examine wound healing at a histological level. The first involved utilizing techniques used in plastic surgery like skin grafts and flaps [35,36,40–44]. These experiments often raised large flaps or grafts that would not be expected to 100% survive. They then use the surface area of the surviving flap/graft as a proxy for how well tissues heal on the assumption that if the tissues heal faster, they will acquire a new blood supply from the surrounding tissues and survive.

The second approach used involved creating a cutaneous wound of specific depth and size and it is monitoring its size over time [23–34,37–39,51]. The rate of re-epithelisation was often calculated in these studies. The histological data obtained in all of these experiments were the secondary outcome to the clinical endpoint of the wound healing or the flap/graft surviving.

### Effect of ADSC on angiogenesis

#### During the first 7 days

**Figure 1.**
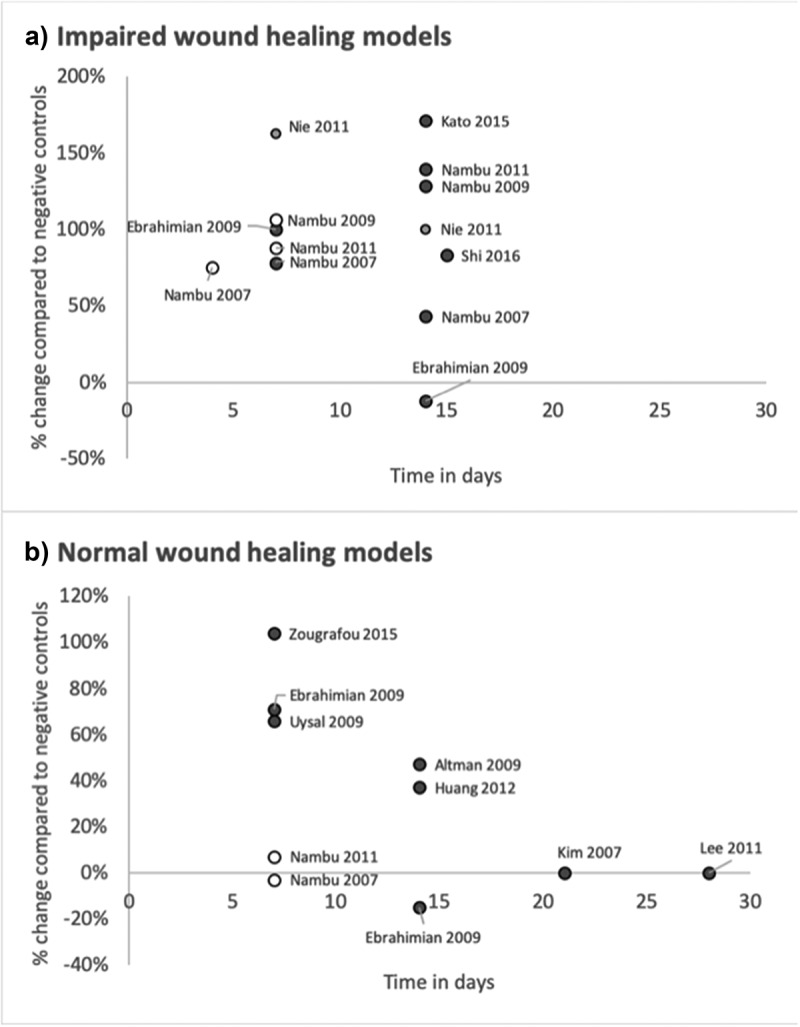
(a) and (b). The percentage change in vessel density at histology with the application of adipose-derived stem cells compared to negative controls at histology. Each point is a separate data point extracted from a study. Grey points show accelerated wound healing at a clinical level and white points were when the clinical change could not be assessed (due to the wound often being splinted open to prevent contraction)

In animals with impaired wound healing, the application of ADSC caused significantly increased angiogenesis compared to negative controls in all studies at 7 days [23–25,31–34] (see figure 1(a)). There was remarkably little variation between different studies results, reporting differences of 70%[25] and 106%[32] compared to negative controls. This was regardless of the method of impairing the healing, which varied significantly from genetic [23,32,33], to chemically [31,34] or with radiation[25]. Additionally, all of these studies reported clinically observable accelerated wound healing.

In healthy animal subjects, the effect of ADSC on angiogenesis at 7 days was more varied; two studies showed no difference in vessel density compared to negative controls [31,33] and three studies showing significantly increased vessel density [25] of between 66%[35] and 104%[36] (see figure 1(b)). One explanation for why ADSC proportionally increase angiogenesis more in impaired wound healing, is that healthy animals already heal efficiently, so have little room for improvement. Evidence for this is provided in one study where the actual number of vessels was reported. Here it was shown that diabetic mice treated with ADSC only surpassed the density of vessels between 7 and 14 days that a non-diabetic mouse reached in a week[33]; the healthy animals are already reaching very high levels of angiogenesis without ADSC, hence why the proportional change is less or sometimes zero.

#### From 8 days onwards

In animals with impaired wound healing, ADSC continue to increase angiogenesis up to 15 days compared to negative controls [23,31–34] in all bar one study[25]. This was seen to a much lesser extent in healthy animals [25,26,28] (three studies less than 50% compared to three studies more than 100%), likely due to angiogenesis reaching a maximal level by this point (see figure 1).

Two studies examined changes in blood vessel density for longer than 14 days and showed no difference compared to negative controls [29,30]. Besides the angiogenesis already reaching a maximal point, another explanation for this observation is that the ADSC would have been vascularized by this point or non-viable, both of which would not affect further angiogenesis.

Additionally blood vessels were noted to be of larger diameter in ADSC-treated wounds compared to narrower controls with haematoxylin and eosin staining (H&E) in one study[34].

### Effect of ADSC on wound healing at a clinical level

At a clinical level, 13 of the 14 studies showed significantly decreased cutaneous wound size with the addition of ADSC ranging between 14% and 47% compared to controls (figure 1) with just a single study using healthy pigs and partial thickness wounds finding no improvement with ADSC[27]. This effect was maintained when efforts were made to stop the wound contracting through splinting (with silicone ‘doughnuts’) [24,30,31] which is particularly important in rodents who unlike humans heal 90% of their wounds via contraction[52]. Wound healing is a three-dimensional process and besides increased rate of re-epithelisation, ADSC-treated wounds showed increased granulation tissue [24,28,31–34], likely leading to a thicker dermis [23,30], epidermis [27] and collagen deposition/alignment[34]. ADSC also increased cell proliferation and decreased apoptosis[34].

### Effect of PRP on fat graft histology

Two studies reported the addition of PRP increased the organization of grafts [46,53] which was shown through photographs of histology with more regular cell distribution than controls. While their photos did clearly show this correlation, it is difficult to quantify this effect and no statistics were reported. Irrespective, however, this does provide some evidence for the fibrin component acting as a scaffold for the grafted cells, which may further contribute to enhanced graft survival. Another hypothesis underlying this observation is that the higher density of cells makes the cells appear “organized’, when in reality they are simply very dense due to a higher survival rate from the PRP and this density gives the illusion of organization.

### Effect of PRP on cutaneous tissue histology

PRP caused increased angiogenesis [38,40,43,44], increasing vessel density by 60%[44] to 260%[42] at between 7 and 14 days. Additionally, several studies demonstrated higher expression of angiogenic growth factors, which can be found in the α-granules of platelets after the injection of PRP [42,44]. PRP also caused increased thickness of granulation tissue[44]. These effects are similar to those of ADSC.

One small porcine study (n = 2 pigs, 22 wounds per pig) found no change at a microscopic level to vascularity, collagen deposition or collagen alignment with the addition of PRP to wounds in healthy pigs[39]. This may be due to their methodology where the PRP was activated for an hour, then re-centrifuged and the fibrin clot removed before being frozen at −80°C until its use. In contrast other studies that reported positive results used their PRP fresh and did not remove the platelet portion [40,41].

### Effect of PRP on fat graft viability

The presence of non-viable tissue in a chronic wound is widely known to be detrimental to spontaneous healing, and debridement with removal of all foreign material is one of the most basic principles of wound healing. It is therefore of critical importance that a high proportion of the grafted fat survive, so as not to hinder the healing process. When fat (containing adipocytes and ADSC) is grafted into healthy cutaneous tissue without PRP, the histological changes were examined in several studies [14,54,55]. Initially, the grafted adipocytes and ADSC become damaged due to the mechanical forces applied during harvest and injection. On day 1, fat grafts were infiltrated by immunological cells (neutrophils then macrophages, histocytes and giant cells), which clear cellular debris while a proportion of adipocytes necrose. On day 4 there was graft vascularization through neo-angiogenesis in the peripheries of the graft. Vacuoles and oily/fatty cysts (signs of ischaemia in adipocytes) were not seen in central adipocytes after day 8. More recently, it was shown in mice that adipocytes located more than 300 μm from the periphery of the fat graft become non-viable in just 1-day [14] as demonstrated by reduced Perilipin staining. Eto et al also showed that standard stains such as H&E are not able to morphologically discriminate between living, dead and dying adipocytes in the first few days (due to the thin microscope slices (5 to 10 μm) compared to large adipocyte cell size (50 to 150 μm), neither the presence of a nucleus or the shape of the cell can be used as a surrogate marker of cell health) [14] which may explain the discrepancy between their findings and previous work. Adipocytes becoming non-viable in just 1-day is much earlier than previously reported, and if true, means that any substance aiming to increase fat graft survival will need to work rapidly.

Additionally, Eto et al demonstrated that after day-7, there is a resurgence in viable adipocytes located just further than 300 μm from the periphery of the graft (approximately another 50 μm into the graft) and markers of cellular proliferation such as Ki67 are increased[14]. The authors postulated this was due to the survival of ADSC in this area that then multiplied and differentiated to produce the Perilipin staining (showing new adipocytes) observed. This was termed the ‘three zone’ theory of fat graft survival made up of:

1) Surviving zone on the peripheral 300 μm of the graft where adipocytes and ADSC survive.

2) Regenerating zone between 300 and 350 μm where adipocytes die but ADSC survive and replenish them after 7 days.

3) Necrotic zone more than 350 μm into the centre of the graft where all cells die, no regeneration is expected, and the area is replaced by scar tissue or resorbed by the body.

**Figure 2.**
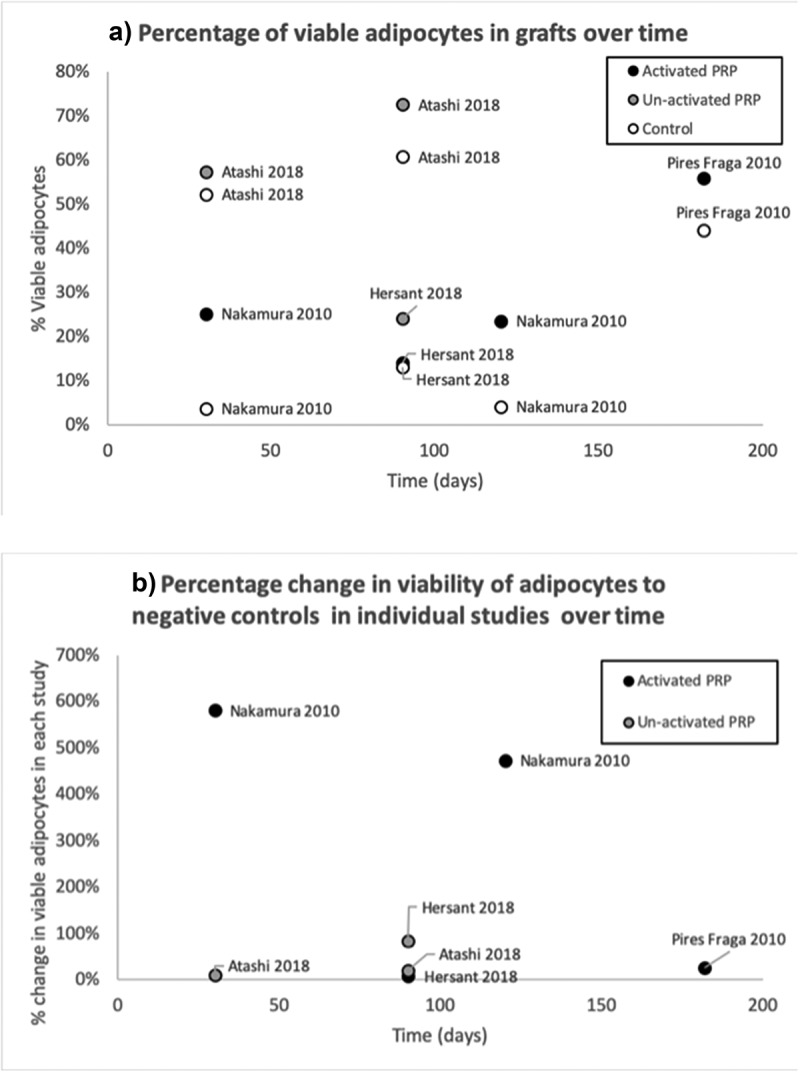
(a) and (b). Effects of platelet-rich plasma on fat graft cell viability from different studies over time. Each point is from data extracted from a different animal study. 2A gives the percentage of viable cells from the graft and 2B shows the percentage change compared to the negative control in that study. Black points show activated platelet-rich plasma, grey is inactivated and white is controls. PRP = platelet-rich plasma

While six studies have reported the changes in volume of fat graft survival with the addition of PRP [10,11,45,47,53,56] this outcome is largely irrelevant to wound healing. Instead, the viability of cells that can then contribute to regeneration and repair is much more important. Across four studies, the application of PRP increased adipocyte viability [10,45–47] (see figure 2). As previously stated, graft re-vascularization needs to occur fast for the adipocytes and ADSC to survive. These animal studies were not designed to identify changes within the first 72 hours, instead of focusing on the long-term viability of cells at 3 to 6 months. Only one study performed histology at 10 days and they did not report their results at this time point in terms of adipocytes viability[47]. Logically, however, long-term cell viability depends upon initial re-vascularization, so any time after 7 days when non-vascularized cells would become hypoxic and die may reasonably be taken as a proxy. Further two studies reported other indirect measures of increased viability [11,53] like decreased vacuoles with PRP compared to just one study showing no difference[56].

Generally activating the PRP had a greater positive effect on mean cell viability than inactivated PRP (see figure 2(b)). In Hersant’s study[46], activated and un-activated PRP were directly compared and they showed activation of PRP with calcium chloride significantly increased mean fat graft viability to 24%, up from 13% and 14% in saline-treated and inactivated PRP treated fat grafts, respectively (see figure 2).

Fat grafts that were applied with PRP were observed to have increased blood vessel density by 91% to 97% [10,11]. This is less relevant to wound healing; however, as cell viability is the important outcome of interest. It is unclear if the PRP or ADSC are responsible for the increased blood vessel density and additionally it is unclear from the data whether this change would have happened within the short time window to prevent graft ischaemia. PRP, which is known to affect angiogenesis may be distorting the results and causing increased neo-angiogenesis but after the critical time for re-vascularization of the fat graft, causing higher vascularity at a later time point, yet with reduced fat graft cell viability. It is important to remember that in animal models, mainly a non-isogenic fat graft (human fat grafted to mice/rat) has been used, and this may cause graft rejection which could consequently explain the bad survival rate after 30 days.

### Combined effect of PRP and ADSC on histology

To date, only one study has examined ADSC and PRP applied jointly to accelerate wound healing. This was performed in healthy pigs and while histology showed significantly increased angiogenesis compared to PRP/ADSC in isolation and negative controls, there was no observable clinical improvement in re-epithelisation[50]. Similar to the finding in other healthy animals previously discussed, it is likely that the pig’s innate healing ability was sufficient to mask any effect of either the ADSC or PRP were having on a clinical level.

## Discussion

From the animal studies presented within this review, it appears that activated PRP enhances the proportion of viable cells in fat grafts [10,11,45–47,57], and increases the microscopic organization of grafts, which is a prerequisite for them to participate in accelerating wound healing [46,53]. Angiogenesis was increased by ADSC and PRP in isolation, and together between 7 days and 3 months. Most studies reported improved or accelerated wound healing at a clinical level and animal studies using models of impaired wound healing often found a greater effect with the application of ADSC/fat grafts and PRP than in healthy subjects [23,24,32–34]. (See Table 1.) Chronic wounds are a growing problem, of which there is an urgent need for novel, efficacious, evidence-based treatments and this review provides in vivo evidence of the efficacy of ADSC and PRP in the setting of wound healing. Their application clinically may improve the management of diabetic foot ulcers.

The fundamental issue of low fat graft survival hinders its clinical use in all settings. Recent evidence has shown that fat grafts needs to be injected very thinly, as mature adipocytes more than 300 μm from the periphery of the graft will become ischaemic and die[14]. Any pro-survival mechanism will need to act very fast, within 1 day, if adipocytes and ADSC located further into the graft are to remain viable. Increased survival of fat grafts with PRP is likely through the uncoordinated release of growth factors from the platelets which increases the rate of initial angiogenesis [15] and prevents graft loss through hypoxia. PRP may additionally improve graft survival through other means; however, this initial early revascularisation is logically the most important step, as without the minimum oxygen requirements met of the cells, the concentration of growth factors or cytokines is irrelevant to the cell’s survival and proliferation. Angiogenesis is reduced in diabetic individuals, another factor which prevents diabetic foot ulcers from healing spontaneously. By amplify angiogenesis, through the application of ADSC and PRP, this treatment addresses one of the key underlying issues in their wound healing.

One concern with the angiogenesis data is the high likelihood of reporting bias. Studies often reported angiogenesis at a one or two time points, but performing histology on animals at multiple other time points [23–25]. During our review process, these authors were contacted for the remaining data yet no further data were provided, as authors were either uncontactable or did not respond. Only one study did not report on vessel density and this was in a partial thickness wound, presumably as the wound was not deep enough into the dermis to damage the vasculature[27].

PRP has a very broad definition (‘platelet concentration above plasma’) [19] and preparations made from different commercial methods are significantly different in their constituents (a greater than fourfold difference in the concentration of platelets has been demonstrated)[58]. Additionally, activation of PRP was variable between studies, and this has been shown to affect both the make-up, and rate of growth factors released[20]. Some studies did not activate their PRP[48]. Finally, the concentration of PRP applied has different effects on cells, and some high concentrations (e.g. 20%) in vitro paradoxically fail to cause ADSC to proliferate[59]. In combination, all of the above factors create a further layer of complexity, which makes direct comparison of studies in this review more challenging.

Despite the apparent efficacy of ADSC of accelerating wound healing and angiogenesis in animals with impaired wound healing, this may not translate into human medicine. The ADSC found in the fat grafts of patients with diabetic ulcers have less regenerative potential as diabetes, advancing age and higher body mass index all reduce the proliferative and differentiation potential of ADSC[60]. Some studies did use ADSC from genetically diabetic mice (*db/db*) [32,33] and still showed accelerated angiogenesis and clinically hastened wound healing. However, despite these mice being severely hyperglycaemic, they share few other clinical findings of the syndrome of diabetes mellitus. It is difficult to postulate to what extent this finding in 10-week-old mice with a normal body habitus translates to humans. Superior diabetic models used included Zucker diabetic fatty rats (*fa/fa*) who besides hyperglycaemia, share more characteristics with human type 2 diabetes than *db/db* mice such as obesity [61] and microvascular complications like kidney disease [62] and peripheral neuropathy[63]. A study using this model also found strongly positive results on a histological and clinical level [23] so these studies may be reaching the limit of what we can learn from animal models. Finally, it is important to remember all of the above studies used ADSC which are at most only 1% of a fat graft, with the other 99% being mostly mature adipocytes with less regenerative potential.

Under physiological conditions ADSC differentiate into adipocytes[64], which are mesodermal in origin. Furthermore, it is accepted that ADSC can differentiate into other cells of mesodermal lineages such as bone [65,66] and cartilage [67,68]. There is weaker evidence that ADSC are capable of differentiating into cells of ectodermal [24,33] and endodermal [23,24,33,36] lineage. One problem within the field of stem cell research is the difficulty in ensuring cell populations are exactly as described. Cell markers are a proxy for a cell type and regardless of the similarities between the cell markers of cell populations and ADSC, there is no guarantee that cells which exhibit a particular set of antigens is what we believe it is. As the cells become more distant from adipocytes, it is the authors opinion that the likelihood of the starting population being purely ADSC reduces.

This review highlights the multiple animal studies that have shown PRP and ADSC in isolation may augment wound healing. However, the quality of these studies is inconsistent, as many have small sample sizes [37,38], inconsistent methodologies and little or no blinding. The use of ADSC in clinical practice is extremely limited due to legislation, both in the UK and worldwide, preventing stem cell use, and hence why fat grafting without further processing is more widely utilized clinically. Further evidence of the efficacy of this treatment in clinical trials is required to definitively answer if this could be the basis of a novel, autologous treatment for diabetic ulcers.

## Supplementary Material

Supplemental Material

## Data Availability

Included as appendix 1.
