# Supplementary material for: Fat grafting and platelet-rich plasma in wound healing: a review of histology from animal studies
Source: Adipocyte. 2021 Feb 2;10(1):80–90. doi: 10.1080/21623945.2021.1876374 (PMC7872055; doi:10.1080/21623945.2021.1876374)
Supplement: Supplemental Material [file KADI_A_1876374_SM3217.docx]

Supplementary material

**Table 1**: in vivo studies examining the effect of adipose-derived stem cells (ADSC) on cutaneous tissue

| **Studies directly examining wound healing** | | | | | | | | | |
| --- | --- | --- | --- | --- | --- | --- | --- | --- | --- |
| **Study** | **Animal model and number** | **Delivery method of ADSC** | **Control** | | **Method of examining wound healing** | | **Microscopic outcome** | **Histo / immunohisto-chemical**  **stains used** | **Clinical outcome** |
| Altman et al. 2009^1^ | Athymic mice.  n=17 mice in total, n=5 for 3 different interventions and 2 for histology. | - Silk fibroin-chitosan scaffold seeded with ADSC. | - Unseeded silk fibroin-chitosan scaffold. - No graft. | | 6mm diameter full-thickness wound. | | - Increased vessel density by 47% at 14 days in ADSC* | - H&E - Von Willebrand factor - Smooth muscle actin - GFP - Ki67 - Heat shock protein 47 | Wound size reduced by 14% more at 6 days in ADSC* |
| Ebrahimian et al. 2009^2^ | C57BI/6 mice.  n= 48 in total, n=6 per group where 5 groups were irradiated (20 Gy) and 3 were not. | - ADSC injected IV or IM in nearby muscle. | - PBS injected IV or IM. | | 8mm diameter full-thickness wounds. | | - Increased average number of vessels by 46-89% at day 7 in non-irradiated mice with addition of IV or IM ADSC* - Increased average number of vessels by 66-80% at day 7 in irradiated mice, with addition of ADSC (IV or IM)* | - H&E - CD31 | Non-irradiated wounds healed 15-18% more at 10 days with addition of ADSC (IV or IM)*  Irradiated wounds healed 32-35% more at 14 days with addition of ADSC (IV or IM)* |
| Hanson et al. 2016^3^ | Göttingen minipigs.  n=8 pigs and 5 wounds per pig. | - ADSC injected ID | - Saline injected ID | | 20x20mm partial thickness wounds. | | - Increased epidermal thickness at 10 days in ADSC* | - H&E | No difference in wound size.  All wounds (control and ADSC) healed by 21 days. |
| Huang et al. 2012^4^ | Nude mice.  n=20 mice in total, n=5 for 4 different interventions | - ADM seeded with ADSC. - ADSC injected into seam between silicone and wound. | - ADM - Silicone only. | | 8mm diameter full-thickness wounds. | | - Increased vessel density by 37% in ADSC + ADM at 14 days* - Increased granulation tissue in ADSC + ADM | - H&E - Von Willebrand factor - VEGF | Wound size reduced by 20% more at 9 days in ADSC* |
| Kato et al. 2015^5^ | Zucker diabetic fatty rats.  n=48 rats in total.  n=24 ADSC  n=24 control | - ADSC sheet overlaid by artificial skin | - Artificial skin alone. | | 10x15mm full-thickness wound. | | - Thicker dermis in ADSC. - Increased vessel density by 171% at day 14 in ADSC* - ADSC were observed in perivascular regions and incorporated into new blood vessels. | - H&E - CD31 - EGFP | Decreased wound size at 7, 10, 14, 18 and 21 days in ADSC*  Decreased mean time to wound closure by 25% in ADSC* |
| Kim et al. 2007^6^ | Nude mice.  n=6 mice in total. 2 wounds per mouse (ADSC + control) | - Collagen gel containing ADSC. | - Collagen gel. | | 7mm diameter full-thickness wound. | | - No difference between groups at 21 days. | - H&E | Wound size reduced by 34% more at 7 days in ADSC* |
| Lee, Lee, and Cho. 2011^7^ | Nude mice.  n=8 mice in total.  n=4 ADSC  n=4 control | - Collagen gel containing ADSC. | - Collagen gel. | | 15mm diameter full-thickness wound, prevented from contracting by splinting. | | - Thicker dermis in ADSC by 63% at 28 days* | - H&E | Wound size reduced by 47% more at 10 days in ADSC* |
| Nambu et al. 2007^8^ | Fisher 344 rats.  n=12 rats in total, 2 wounds per rat. Mitomycin C washed over wounds. | - ADSC + ACMS to mitomycin C washed wounds | - ACMS alone to mitomycin C washed wounds - Skin wounds only | | 15mm diameter full-thickness wounds, splinted for 7 days to prevent contraction. | | - Increased vessel number by 43-85% at 4, 7 and 14 days in ADSC treated wounds* - Increased granulation thickness by 42-85% at 4, 7 and 14 days in ADSC treated wounds* | - H&E | Decreased wound size in ADSC treated wounds after removal of splint at 7 days* |
| Nambu et al. 2009^9^ | db/db mice.  n= 24 mice in total, 2 wounds per mouse. | - ADSC containing ACMS | - ACMS alone. | | 15mm diameter full-thickness wounds. | | - Increased granulation thickness by 174% at 14 days in ADSC group* - Increased vessel density by 128%* at 14 days in ADSC group• | - H&E | Wound size reduced by 30% more at 14 days with ADSC* |
| Nambu et al. 2011^10^ | db/db mice.  n=12 in total. | - ADSC in ACMS | - ACMS alone. | |  | | - Granulation tissue thickness increased by 178% at 14 days in ADSC* - Vessels increased by 177% at 14 days in ADSC* - ADSC incorporated into granulation and epithelial tissues. - Some ADSC differentiated into vessels and endothelium | - H&E - CD31 | Wound size reduced by 35% at 14 days in ADSC* |
| Nie et al. 2011^11^ | Lewis rats.  n=72 rats in total. n=36 were made ‘diabetic’ by injecting streptozotocin  2 wounds per rat | - ADSC in PBS injected ID | - PBS injected ID | | 8mm diameter full-thickness wounds, splinted to prevent contraction. | | - Increased vessel density by 99% at 14 days in ADSC* - Increased total granulation tissue at 7, 10 and 15 days in ADSC* - ADSC were incorporated into dermal structures. - ADSC were incorporated into endothelial cells. - Increased expression of FGF2 in ASDC wounds* | - H&E - GFP - CD31 - Pan-cytokeratin | Decreased mean time to wound closure by 36% in ADSC in diabetic rats*  Decrease mean time to wound closure by 27% in ADSC group in normal rats. |
| Shi et al. 2016^12^ | Sprague-Dawley rats.  n=36 rats in total,  n=18 ADSC  n=18 control.  All rats made diabetic’ by injecting streptozotocin | - ADSC injected IV | - PBS injected IV | | 5mm diameter full-thickness wound. | | - Granulation tissue was 47% thicker at 15 days in ADSC* - Increased density of collagen deposition by 108% in ADSC* - Collagen more organised and aligned in ADSC - Increased vessel density by 75% at 15 days in ADSC* - Increased cell proliferation by 244% at 15 days in ADSC* - Decreased cell apoptosis by 38% at 15 days in ADSC* - Larger vessels in ADSC wounds. - ADSC injected IV were able to migrate to a wound and participate in wound healing* | - H&E - Masson’s trichrome - TUNEL - Ki67 - CD31 - ZsGreen | Wound size decreased by 21% more at 7 days and 41% more at 15 days in ADSC* |
| **Studies indirectly examining wound healing through ‘plastic surgical’ techniques** | | | | | | | | | |
| Uysal et al.2009^13^ | ICR mice.  n=20 mice in total, 2 flaps per mouse. | - Flap injected with ADSC. | | - Flap injected with PBS | Cranial based random flaps of size 1x5cm^2^ raised and then pedicle clamped for 6 hours. | - Increased average number of vessels by 133% in ADSC flaps* - Increased vessel density by 66% in ADSC flaps* - Increased endothelial cells by 137% in ADSC flaps* - Increased VEGF expression by 98% in ADSC flaps* - Increased FGF expression by 69% in ADSC flaps• - Less inflammation in ADSC flaps. - Increased collagen content in ADSC flaps. | | - H&E - Von Willebrand factor - VEGF - TGF-β1 - TGF-β2 - TGF-β3 - FGF - DiI | Increased flap survival at 7 days by 69% surface area* |
| Zografou et al. 2011^14^ | Sprague-Dawley rats.  n=20 | - ADSC injected into recipient graft bed | | - PBS injected into recipient graft bed | 3x3cm^2^ full-thickness skin removed and then grafted into old defect. | - Increased collagen by 31% at 7 days in ADSC grafts* - Increased vessel density by 104% at 7 days in ADSC grafts* - Increased VEGF expression by 15-18% in graft bed and epidermis-dermis at 7 days in ADSC group* - Increased TGF-β3 expression by 10-16% in graft bed and epidermis-dermis at 7 days in ADSC group* - DiI labelled cells (from ADSC) observed in newly formed capillaries | | - H&E - Von Willebrand factor - VEGF - Masson’s trichrome - TGF-β3 - DiI | Graft survival increased by 26% in ADSC group* |

** = significant result (p<0.05). Abbreviations used are ADSC = Adipose-derived stem cells, PBS = phosphate-buffered saline, ADM = acellular dermal matrix, IV = intravenous, IM = intramuscular, ID = intradermal, H&E = haematoxylin and eosin, TUNEL = Terminal deoxynucelotidyl transferase dUTP nick end labelling, ACMS = atelocollagen matrix with silicone membrane. All percentages rounded to nearest whole number.*

**Table 2**: in vivo studies examining effect of platelet-rich plasma (PRP) on cutaneous tissue

| **Studies directly examining wound healing** | | | | | | | |
| --- | --- | --- | --- | --- | --- | --- | --- |
| **Study** | **Animal model and number** | **Delivery method of PRP** | **Control** | **Method of examining wound healing** | **Microscopic outcome** | **Histological / immunohisto-chemical**  **stains used** | **Clinical outcome** |
| Carter et al. 2003^15^ | Thoroughbred horse.  n=1 horse, 14 wounds. | - PRP gel. | - Saline | 2.5cm^2^ full-thickness wounds. | - Thinner epithelial layer by 50% in control wounds at day 79. - Increased differentiation of cells in PRP group. - More organised collagen fibres in PRP wounds | - H&E - Cytokeratin 10 - Masson’s trichrome |  |
| DeRossi et al. 2009^16^ | Saddle horse.  n=6 horses in total, 2 wounds per horse. | - PRP gel to subcutaneous tissue e before sutured. | - Wound just sutured | Full-thickness excisional wounds re-sutured. | - Less mononuclear inflammatory infiltrate in PRP wounds - Absent epithelial covering in control group - More vessels in PRP group at 30 and 45 days. | - H&E - Masson’s trichrome | Small are of ulceration in control group. |
| Long et al. 2017^17^ | Yorkshire pigs.  n=2 pigs.  22 wounds per pig | - PRP into wounds | - Saline | 2cm diameter full-thickness wounds. | - No difference in vascularity, collagen deposit or collagen alignment. | - H&E - Masson’s trichrome - Picrosirius red - Von Willebrand factor - Cytokeratin | No difference in re-epithelisation rate |
| **Studies indirectly examining wound healing through ‘plastic surgical’ techniques** | | | | | | | |
| Findikcioglu et al. 2012^18^ | Rats  n=29 rats in total, 2 flaps per rat.  n=8 PRP  n=8 fibrin glue  n=8 thrombin | - PRP sprayed under flap | - Nothing under flap | Epigastric axial pattern flaps raised and pedicle ligated after 5 days. | - Increased re-epithelisation, neo-vascularisation, and collagen formation at 7 days after ligation in PRP flaps* - Decreased inflammatory infiltration in PRP flaps at 7 days after ligation* | - H&E - VEGF - TGF-β3 - PDGF | Increased flap survival by 10% at 7 days after ligation in PRP flaps* |
| Kim et al. 2013^19^ | New Zealand rabbits.  n=16 rabbits in total. | - Flap injected SC with PRP | - Flap injected with saline | 10x10cm unipedicled fasciocutaneous island flap | - Micro-vessel density increased by 120% at 14 days in PRP flaps* - Mature vessel density increased by 120% at 14 days in PRP flaps* | - H&E - CD31 - Smooth muscle | Flap survival increased by 45% at 14 days in PRP flaps* |
| Li et al. 2012^20^ | Wistar rats.  n=48 rats in total.  n=16 PRP  n=16 PPP  n=16 no treatment | - PRP injected SC into flaps | - PPP injected SC into flaps - No treatment | 11x3cm cranially based dorsal cutaneous flaps | - Decreased inflammatory cells by 77% at 7 days in PRP group* - Increased blood vessel density by 260% at 7 days in PRP group* - Increased expression of VEGF and PDGF mRNA* | - H&E - Von Willebrand factor | Increased flap survival by 71% at 7 days in PRP group* |
| Sönmez et al. 2013^21^ | VEGFR2-luc mice.  N=24 mice in total. | - PRP gel - PRP gel + ischaemia | - Sham operation - Ischaemia | 1.5x2.5cm axial pattern island flap. | - Increased vessel density by 133% at 14 days in PRP + ischaemia* - No difference in vessel density in PRP alone at 14 days | - H&E - Von Willebrand factor - VEGF | Increased flap survival by 40% at 14 days in PRP + ischaemia*  Increased flap survival in PRP alone |
| Takikawa et al. 2011^22^ | Fisher 344 rats.  n=52 rats in total.  Two flaps per rat.  n=8 per group | - PRP injected ID into flaps either alone or with F/PMP | - PBS injected ID into flaps | 8x2cm caudally based random pedicle flaps | - Increased mean vessel number by 60% at 7 days in PRP alone flaps than controls* - Increased granulation thickness in PRP flaps at 7 days. - Increased expression of VEGF at 7 days in PRP injected flaps. | - H&E - VEGF | Increased flap survival by 16% at 7 days when PRP + F/PMP injected 2 days before raising flap*  No difference between PRP and controls when injected just as flap raised, 2 hours before or 1 day before. |

** = significant result (p<0.05). Abbreviations used are PRP = platelet-rich plasma, PPP = platelet-poor plasma, PBS = phosphate-buffered saline, F/PMP = fragmin/protamine microparticles, IV = intravenous, IM = intramuscular, ID = intradermal, SC = subcutaneous, H&E = haematoxylin and eosin. All percentages rounded to nearest whole number.*

**Table 3**: in vivo studies examining effect of platelet-rich plasma (PRP) on fat graft survival

| **Study** | **Animal model and number** | **Adipose harvest and grafting** | **Form of PRP** | **Control** | **Microscopic outcome** | **Histo. / immunohisto-chemical stains used** | **Clinical outcome** |
| --- | --- | --- | --- | --- | --- | --- | --- |
| Atashi et al. 2019^23^ | Nude mice (Balb/cAnNRj). | - Harvested from human abdominoplasty tissue - Grafted into the scalp of mice | - Not activated - 20% | - Fat graft with saline | - Increased fat graft area occupied by intact adipocytes in inactivated PRP group compared to controls at 3 months* - Increased vascularity in inactivated PRP compared to controls by 58% at 3 months* | - H&E - Vimentin - CD31 - Perilipin - Hoechst | No difference in fat graft volumes at 1, 2 or 3 months |
| Hersant et al. 2018^24^ | Nude rats.  N=8 rats in total. Each rat received 3 fat grafts (control, not activated and activated) | - Harvested from human abdominoplasty tissue - Grafted into flank and inferior spinal region of rats | - Not activated - Activated with CaCl_2_ | - Fat graft with saline | - Less fibrous bundles with reduced thickness in activated PRP compared to inactivated PRP and controls at 3 months - Decreased macrophage infiltration in activated PRP group - Similar pericellular fibrosis in all 3 groups - Similar vascularity at 3 months in all groups | - H&E - Perilipin - CD45 - CD31 | Activated PRP fat grafts contained mean viable adipocytes of 24% compared to 14% in inactivated and 13% in controls* (71% increase in viable adipocyte area) |
| Nakamura et al. 2010^25^ | Fisher 334 rats.  n=64 rats in total. | - Harvested from inguinal region - Grafted into subcutaneous dorsal pocket | - Activated with CaCl_2_ | - Fat graft alone | - Increased vessel formation at 20, 30* and 120* days in PRP group - Increased normal adipocytes* and less ruptured adipocytes at 20 and 30 days in PRP | - H&E - Zudan III | Increased graft survival at 30 and 120 days in PRP*  Fat grafts with PRP had soft, supple feel ad were easily compressible |
| Oh et al. 2011^26^ | Nude mice.  n=20 mice in total.  n=10 PRP  n=10 control | - Harvested via Coleman from healthy woman - Injected SC | - Activated with thrombin and CaCal_2_ | - Fat graft with saline | - Increased vessels by 91% at 10 weeks in PRP* - Less oily cysts and vacuoles (ischaemia/necrosis) at 10 weeks in PRP* - Less fibrosis at 10 weeks in PRP* - No difference in cellular integrity - No difference in inflammation | - H&E - CD31 | Increased fat graft weight and volume by 14% and 25% respectively at 10 weeks in PRP* |
| Pires Fraga et al. 2010^27^ | New Zealand rabbits.  n=30 rabbits in total. | - Harvest via scissor dissection from scapula area - Grafted as 10mm blocks into ear | - Activated with CaCl_2_ - 10:1 ratio | - Fat graft alone | - Increased number of blood vessels by 97% at 6 months in PRP* - Increased viable adipocytes by 27% at 6 months in PRP * - Decreased number of necrotic areas by 35% at 6 months in PRP* - Lower degree of fibrosis at 6 months in PRP* | - H&E | Greater fat graft weight at 6 months in PRP* |
| Por et al. 2009^28^ | Nude mice.  n=24 | - Harvest via Coleman from discarded abdominoplasty tissue - Injected SC into scalp | - Not activated. - Platelet count of 280 x 10^9^ | - Fat graft with saline | - No difference in vascularity, number of cysts, fibrosis, necrosis or inflammation | - Oli Red O | No difference in graft weight or volume between groups |
| Rodríguez-Flores et al. 2011^29^ | New Zealand rabbits  n=9 rabbits in total.  n=8 | - Harvest via modified Coleman from groin pads - Injected in upper lip | - Activated with CaCl_2_ | - Fat graft injected alone | - Reduced inflammatory reaction at 8 and 12 weeks with PRP* - Reduced oil cysts (ischaemia/necrosis) at 8 and 12 weeks with PRP* - No difference in number of blood vessels. - Less fibrosis at 8 and 12 weeks with PRP - Grafted adipocytes more organised with PRP. | - H&E | No difference in quantity of fat graft survival |

** = significant result (p<0.05). Abbreviations used are PRP = Platelet-rich plasma, CaCl_2_ = Calcium chloride, H&E = haematoxylin and eosin. All percentages rounded to nearest whole number.*

References

1. Altman AM, Yan Y, Matthias N, et al. IFATS collection: Human adipose-derived stem cells seeded on a silk fibroin-chitosan scaffold enhance wound repair in a murine soft tissue injury model. *Stem Cells*. Jan 2009;27(1):250-8. doi:10.1634/stemcells.2008-0178

2. Ebrahimian TG, Pouzoulet F, Squiban C, et al. Cell therapy based on adipose tissue-derived stromal cells promotes physiological and pathological wound healing. *Arterioscler Thromb Vasc Biol*. Apr 2009;29(4):503-10. doi:10.1161/ATVBAHA.108.178962

3. Hanson SE, Kleinbeck KR, Cantu D, et al. Local delivery of allogeneic bone marrow and adipose tissue-derived mesenchymal stromal cells for cutaneous wound healing in a porcine model. *J Tissue Eng Regen Med*. Feb 2016;10(2):E90-E100. doi:10.1002/term.1700

4. Huang SP, Hsu CC, Chang SC, et al. Adipose-derived stem cells seeded on acellular dermal matrix grafts enhance wound healing in a murine model of a full-thickness defect. *Ann Plast Surg*. Dec 2012;69(6):656-62. doi:10.1097/SAP.0b013e318273f909

5. Kato Y, Iwata T, Morikawa S, Yamato M, Okano T, Uchigata Y. Allogeneic Transplantation of an Adipose-Derived Stem Cell Sheet Combined With Artificial Skin Accelerates Wound Healing in a Rat Wound Model of Type 2 Diabetes and Obesity. *Diabetes*. Aug 2015;64(8):2723-34. doi:10.2337/db14-1133

6. Kim WS, Park BS, Sung JH, et al. Wound healing effect of adipose-derived stem cells: a critical role of secretory factors on human dermal fibroblasts. *J Dermatol Sci*. Oct 2007;48(1):15-24. doi:10.1016/j.jdermsci.2007.05.018

7. Lee SH, Lee JH, Cho KH. Effects of Human Adipose-derived Stem Cells on Cutaneous Wound Healing in Nude Mice. *Ann Dermatol*. May 2011;23(2):150-5. doi:10.5021/ad.2011.23.2.150

8. Nambu M, Ishihara M, Nakamura S, et al. Enhanced healing of mitomycin C-treated wounds in rats using inbred adipose tissue-derived stromal cells within an atelocollagen matrix. *Wound Repair Regen*. 2007 Jul-Aug 2007;15(4):505-10. doi:10.1111/j.1524-475X.2007.00258.x

9. Nambu M, Kishimoto S, Nakamura S, et al. Accelerated wound healing in healing-impaired db/db mice by autologous adipose tissue-derived stromal cells combined with atelocollagen matrix. *Ann Plast Surg*. Mar 2009;62(3):317-21. doi:10.1097/SAP.0b013e31817f01b6

10. Nambu M, Ishihara M, Kishimoto S, et al. Stimulatory Effect of Autologous Adipose Tissue-Derived Stromal Cells in an Atelocollagen Matrix on Wound Healing in Diabetic db/db Mice. *J Tissue Eng*. 2011;2011:158105. doi:10.4061/2011/158105

11. Nie C, Yang D, Xu J, Si Z, Jin X, Zhang J. Locally administered adipose-derived stem cells accelerate wound healing through differentiation and vasculogenesis. *Cell Transplant*. 2011;20(2):205-16. doi:10.3727/096368910X520065

12. Shi R, Jin Y, Cao C, et al. Localization of human adipose-derived stem cells and their effect in repair of diabetic foot ulcers in rats. *Stem Cell Res Ther*. 10 2016;7(1):155. doi:10.1186/s13287-016-0412-2

13. Uysal AC, Mizuno H, Tobita M, Ogawa R, Hyakusoku H. The effect of adipose-derived stem cells on ischemia-reperfusion injury: immunohistochemical and ultrastructural evaluation. *Plast Reconstr Surg*. Sep 2009;124(3):804-15. doi:10.1097/PRS.0b013e3181b17bb4

14. Zografou A, Tsigris C, Papadopoulos O, et al. Improvement of skin-graft survival after autologous transplantation of adipose-derived stem cells in rats. *J Plast Reconstr Aesthet Surg*. Dec 2011;64(12):1647-56. doi:10.1016/j.bjps.2011.07.009

15. Carter CA, Jolly DG, Worden CE, Hendren DG, Kane CJ. Platelet-rich plasma gel promotes differentiation and regeneration during equine wound healing. *Exp Mol Pathol*. Jun 2003;74(3):244-55.

16. DeRossi R, Coelho AC, Mello GS, et al. Effects of platelet-rich plasma gel on skin healing in surgical wound in horses. *Acta Cir Bras*. 2009 Jul-Aug 2009;24(4):276-81.

17. Long DW, Johnson NR, Jeffries EM, Hara H, Wang Y. Controlled delivery of platelet-derived proteins enhances porcine wound healing. *J Control Release*. 05 2017;253:73-81. doi:10.1016/j.jconrel.2017.03.021

18. Findikcioglu F, Findikcioglu K, Yavuzer R, Lortlar N, Atabay K. Effect of intraoperative platelet-rich plasma and fibrin glue application on skin flap survival. *J Craniofac Surg*. Sep 2012;23(5):1513-7. doi:10.1097/SCS.0b013e3182597ce6

19. Kim HY, Park JH, Han YS, Kim H. The effect of platelet-rich plasma on flap survival in random extension of an axial pattern flap in rabbits. *Plast Reconstr Surg*. Jul 2013;132(1):85-92. doi:10.1097/PRS.0b013e318290f61b

20. Li W, Enomoto M, Ukegawa M, et al. Subcutaneous injections of platelet-rich plasma into skin flaps modulate proangiogenic gene expression and improve survival rates. *Plast Reconstr Surg*. Apr 2012;129(4):858-66. doi:10.1097/PRS.0b013e3182450ac9

21. Sönmez TT, Vinogradov A, Zor F, et al. The effect of platelet rich plasma on angiogenesis in ischemic flaps in VEGFR2-luc mice. *Biomaterials*. Apr 2013;34(11):2674-82. doi:10.1016/j.biomaterials.2013.01.016

22. Takikawa M, Sumi Y, Ishihara M, et al. PRP&F/P MPs improved survival of dorsal paired pedicle skin flaps in rats. *J Surg Res*. Sep 2011;170(1):e189-96. doi:10.1016/j.jss.2011.05.051

23. Atashi F, André-Lévigne D, Colin DJ, Germain S, Pittet-Cuénod B, Modarressi A. Does non-activated platelet-rich plasma (PRP) enhance fat graft outcome? An assessment with 3D CT-scan in mice. *J Plast Reconstr Aesthet Surg*. Apr 2019;72(4):669-675. doi:10.1016/j.bjps.2018.12.039

24. Hersant B, Bouhassira J, SidAhmed-Mezi M, et al. Should platelet-rich plasma be activated in fat grafts? An animal study. *J Plast Reconstr Aesthet Surg*. 05 2018;71(5):681-690. doi:10.1016/j.bjps.2018.01.005

25. Nakamura S, Ishihara M, Takikawa M, et al. Platelet-rich plasma (PRP) promotes survival of fat-grafts in rats. *Ann Plast Surg*. Jul 2010;65(1):101-6. doi:10.1097/SAP.0b013e3181b0273c

26. Oh DS, Cheon YW, Jeon YR, Lew DH. Activated platelet-rich plasma improves fat graft survival in nude mice: a pilot study. *Dermatol Surg*. May 2011;37(5):619-25. doi:10.1111/j.1524-4725.2011.01953.x

27. Pires Fraga MF, Nishio RT, Ishikawa RS, Perin LF, Helene A, Malheiros CA. Increased survival of free fat grafts with platelet-rich plasma in rabbits. *J Plast Reconstr Aesthet Surg*. Dec 2010;63(12):e818-22. doi:10.1016/j.bjps.2010.07.003

28. Por YC, Yeow VK, Louri N, Lim TK, Kee I, Song IC. Platelet-rich plasma has no effect on increasing free fat graft survival in the nude mouse. *J Plast Reconstr Aesthet Surg*. Aug 2009;62(8):1030-4. doi:10.1016/j.bjps.2008.01.013

29. Rodríguez-Flores J, Palomar-Gallego MA, Enguita-Valls AB, Rodríguez-Peralto JL, Torres J. Influence of platelet-rich plasma on the histologic characteristics of the autologous fat graft to the upper lip of rabbits. *Aesthetic Plast Surg*. Aug 2011;35(4):480-6. doi:10.1007/s00266-010-9640-5
